# Insights into mucosal and systemic immune responses of African catfish, *Clarias gariepinus*, to chilodonellosis: A natural infection study

**DOI:** 10.1111/jfb.70131

**Published:** 2025-06-30

**Authors:** Walaa F. A. Emeish, Salwa Mansour, Marwa M. Fawaz, Ali H. Alghamdi, Abdullah A. A. Alghamdi, Zeinab Al‐Amgad, Haitham H. Mohammed, Catrin S. Rutland, Ahmad A. Elkamel, Karima A. Bakry

**Affiliations:** ^1^ Department of Fish Diseases Faculty of Veterinary Medicine, South Valley University Qena Egypt; ^2^ Zoology Department Faculty of Science, South Valley University Qena Egypt; ^3^ Department of Parasitology Faculty of Veterinary Medicine, South Valley University Qena Egypt; ^4^ Department of Biology, Faculty of Science Al‐Baha University Al‐Baha Saudi Arabia; ^5^ General Authority for Veterinary Services Qena Veterinary Directorate Qena Egypt; ^6^ Department of Rangeland, Wildlife, and Fisheries Management Texas A&M University College Station Texas USA; ^7^ Department of Aquatic Animal Medicine and Management Faculty of Veterinary Medicine, Assiut University Assiut Egypt; ^8^ School of Veterinary Medicine and Science, University of Nottingham Nottingham UK

**Keywords:** ectoparasites, gene expression, *IL‐10*, *IL‐1β*, immune response, *MHC‐II*

## Abstract

A natural parasitic infection with the external ciliate protozoan, *Chilodonella hexasticha*, was recorded 3 days post‐transportation (PT) in the gills and skin of African catfish (*Clarias gariepinus*). Infected fish displayed behavioural changes and typical signs of infection. Mortalities started on day 7 PT and stopped on day 13 PT, where cumulative mortality reached 62.2%. The expression of immune‐relevant genes, interleukin‐1β (*IL‐1β*), major histocompatibility complex class II (*MHC‐II*) and interleukin‐10 (*IL‐10*) in the gills, head kidneys and spleen of the *Chilodonella*‐infected and control uninfected fish were investigated using quantitative real‐time polymerase chain reaction (PCR) on days 7‐, 14‐ and 28 PT. Expression levels of *IL‐1β* in the gills showed a significant upregulation on day 7 PT but were not significantly different from those of the control fish in the head kidneys and spleen at all time points investigated. Expression levels of *MHC‐II* were significantly elevated in the gills (days 7‐ and 14 PT), head kidneys (days 7‐, 14‐ and 28 PT) and spleen (days 7‐ and 14 PT), whereas *IL‐10* showed a significant upregulation only in the gills on day 14 PT, with no significant changes in all other tissues examined. Additionally, histological studies were conducted to investigate the alterations in the head kidney and spleen tissue structures associated with the immune response to infection and the changes in the expression profiles of the immune‐related genes in these organs. Depletion of the renal and splenic tissues, simultaneously with prominent melanomacrophage infiltration, was observed. Also, the histopathologic changes caused by *C. hexasticha* in the gills of infected fish involved aggravated tissue damage characterized by hyperplasia, and necrosis of the gill lamellae was recorded.

## 1 INTRODUCTION

African catfish (*Clarias gariepinus*) stands out as a cornerstone species in the aquaculture industry across many African nations, including Egypt. Its economic significance stems from a host of advantageous traits, notably its rapid growth rate, remarkable adaptability to diverse environmental conditions and capacity to thrive at high stocking densities (Oscar et al., 2015). Despite these advantages, infectious diseases, including parasitic infection, limit the expansion of *C. gariepinus* farming (Abo‐Esa, 2008).

Ciliates are among the most dangerous parasites to fish, and they can lead to secondary bacterial infections (Lom & Dyková, 1992; Pádua et al., 2013). Ciliates of the genus *Chilodonella* (Phyllopharyngea: Chilodonellidae) are primarily free‐living protozoa, but pathogenic species have caused major losses in at least 16 species of freshwater fish (Bradley et al., 2014; Mitra et al., 2013) through parasitizing the skin and gills of fish. Stressful conditions may favour the disease outbreaks by opportunistic pathogens (Danba et al., 2015). Stress is crucial for the development of chilodonellosis and the manifestation of its signs (Bastos Gomes, 2017). Thus, when fish are subjected to adverse conditions, chilodonellosis outbreaks are highly transmissible and can result in high mortalities and great economic losses.

Clinical signs associated with *Chilodonella* spp. infections are not unique. However, fish heavily affected by *Chilodonella* spp. infection may display symptoms such as anorexia, gasping, irritation, skin depigmentation, ulceration and excessive mucous production, resulting in a greyish appearance on both the body and gill lesions (Bradley et al., 2014; Pádua et al., 2013). Severe *Chilodonella* infections can lead to acute gill lesions that are fatal to the affected host (Bowater & O'Donoghue, 2015; Pádua et al., 2013).

Although many fish immune‐related genes have been characterized at the molecular level, scarce data are available about their expression during parasitic infection, especially in relation to *Chilodonella* infections in *C*. *gariepinus*. The head kidney, spleen and mucosa‐associated lymphoid tissues, including the gills, are the primary lymphoid tissues in teleost fishes (Press & Evensen, 1999). Numerous studies have been conducted in several fish species to identify immune‐related genes to gain a better understanding of the molecular immune responses to fish parasites. The most extensively studied *Ciliophora* infection in terms of the expression of immune genes is caused by *Ichthyophthirius multifiliis* in experimentally infected fish (Alvarez‐Pellitero, 2008).

There is a lack of knowledge about the immune responses exhibited by *C. gariepinus* to natural *Chilodonella* infections following transportation stress. This includes which immune genes are expressed in systemic and mucosal immune tissues, as well as whether fish exhibit systemic immune response besides the localized mucosal responses. Consequently, it is vital to understand the mechanisms of immune responses in fish once infected with *Chilodonella* following stress to develop effective mitigation strategies during or after stressful conditions such as those encountered during transportation. The objective of this study was to investigate the expression of certain innate and adaptive immune‐related genes in *C. gariepinus* following a stress‐induced natural infection with the ciliate *Chilodonella*. The relative expression of the proinflammatory cytokine, *IL‐1β*, cell receptor, *MHC‐II* and regulatory cytokine, *IL‐10*, in mucosal (gills) and central immune organs (the head kidneys and spleen) was studied over time to determine whether the immune response to the infection was localized or extended systemically beyond the external sites of infection. Moreover, the histological changes in the central immune organs, resulting from the immune response to the infection, and the changes in gene expression, and the histopathological alterations in the gills due to the active parasitic infection were evaluated.

## 2 MATERIALS AND METHODS

### 2.1 Fish husbandry

The experimental protocols used for this research, including the number of fish and methods, were approved by the Research Bioethics Committee (RBC) of the Faculty of Veterinary Medicine at South Valley University, Qena Province, Egypt, approval no. ‘VM/SVU/23(1)‐09’, and were in accordance with the ARRIVE guidelines (Percie du Sert et al., 2020). Pond‐reared *C. gariepinus* juveniles (*n* = 300), with a body weight ranging from 100 to 150 g, were obtained from a commercial catfish farm, Assiut Governorate, Egypt, and transported for 3 h in fibreglass transport tanks to the aquatic animal housing facility, Department of Fish Diseases, Faculty of Veterinary Medicine, South Valley University, Qena. Fish were transported following routine procedures for transport of experimental fish for in vivo studies in the aquatic laboratory. Upon arrival at the aquatic animal housing facility, following the protocol described by Noga (2010), 10 fish from the stock underwent microbiological and microscopic analysis to screen for and exclude bacterial, fungal and parasitic infections. The remaining fish were equally distributed into 10 fibreglass holding tanks (260 L) in a flow‐through system for quarantine and acclimation to the laboratory conditions prior to use for experimental studies (another unrelated project). The fibreglass tanks were supplied with dechlorinated heated fresh city water. The water quality parameters were maintained as follows: dissolved oxygen 7.0–7.5 mg/L, temperature 27.1°C ± 0.8, pH 7.2–8.4 and ammonia 0.07 ± 0.03 mg/L. Fish were fed 3% of their body weight twice daily with commercial pellets (Skretting, Egypt).

Three days post‐transportation (PT), fish started to exhibit clinical signs of irritation and flashing behaviour due to suspected natural ectoparasitic infestation. Thus, the fish were treated only once with a 2% sodium chloride (NaCl) static bath for 1 h. Because this batch started displaying signs of disease shortly after transportation, another batch of apparently healthy *C. gariepinus* juveniles (*n* = 150) with a body weight range of 110–150 g was obtained from a fish hatchery, the same source of catfish seed used to stock the commercial catfish farm from which the first batch was obtained (3‐h transport). Upon arrival of the second batch in the aquatic animal housing facility, fish were housed in five fibreglass tanks (260 L) in a different system, and 10 fish were screened to rule out infections and diseases. Both groups were treated consistently once introduced to the laboratory. A similar salt treatment was applied to this batch upon arrival at the laboratory. Fish from this batch were used as the uninfected control group. The fish infected (from the first batch) showed the first mortality on day 7 PT.

### 2.2 Experimental design

Out of the fish showing signs of ectoparasitic infection, 60 fish were taken and divided into four groups, each containing 15 fish in four fibreglass experimental tanks (180 L). Groups 1–3 were used to sample fish on 7‐, 14‐, and 28 PT, respectively, whereas the fourth group was used to record the cumulative mortalities and record the clinical signs. Forty‐five fish of the uninfected (control) batch were divided into three groups with 15 fish each and housed in three fibreglass tanks (180 L). Control groups, corresponding to the infected groups 1–3, were sampled at the same time points. The entire experiment was done in triplicate and is schematically represented in Figure S1.

### 2.3 Fish sampling

The onset of the infected fish mortalities occurred on day 7 PT, and therefore it was chosen as the first sampling time point. Fish were killed using 40 mg/L of clove oil (Griffiths, 2000) on 7‐, 14‐ and 28 PT, and samples (*n* = 3) were collected from both infected and control groups for parasitological examination, gene expression analysis and histopathological studies.

### 2.4 Parasitological examination

Standard wet‐mount specimens of gill biopsy (*n* = 3) and skin scraping (n = 3) were collected from both control and infected groups at each corresponding sampling time for parasitological examination. Other skin and gill specimens were fixed with absolute methyl alcohol and stained with Giemsa stain. Examinations of both fresh and stained smears were performed using a Leica digital microscope (Leica Microsystems, Singapore) under low and high objectives and oil immersion lenses according to the methods described by Kabata (1985) and Woodland (2006). A quantitative scoring of the severity of infection in gills at various sampling points was conducted by examining one smear per fish with five fields per smear.

### 2.5 Gene expression analysis

#### 2.5.1 RNA extraction and cDNA synthesis

Approximately 30 mg of gills (*n* = 3), head kidneys (*n* = 3) and spleen (*n* = 3) were dissected from both control and infected fish at each corresponding sampling time point. Total RNA was extracted from tissue samples using RNeasy Mini kit Reagent (Qiagen, Hilden, Germany) following the manufacturer's recommendations. The quality and quantity of the RNA extracts were analysed by measuring the absorbances at 260 (A260) and 280 nm (A280) using the NanoDrop LITE Spectrophotometer (Thermo Scientific, USA). Only high‐quality RNA samples based on A260/A280 ratios with purity of 2.1 ± 0.2 were used for quantitative polymerase chain reaction (qPCR) analysis.

To synthesize the first strand of complementary DNA (cDNA), 1 μg of the total RNA was reverse transcribed by the RevertAid first strand cDNA Synthesis kit (Thermo Scientific) according to the manufacturer's protocols.

#### 2.5.2 Primer design and amplification efficiency

The expression profiles of the three immune‐related genes (*IL‐1β*, *MHC‐II*, *IL‐10*) were assessed using two housekeeping genes, beta‐actin (*β‐actin*) and glyceraldehyde‐3‐phosphate dehydrogenase (*GAPDH*) of *C. gariepinus*. Gene‐specific primers were designed based on sequences with accession numbers available in the GenBank (NCBI), as listed in Table S1. Primers used in the present study were synthesized by Macrogen Humanizing Genomics, Seoul, Republic of Korea. Primer amplification efficiency (E) was calculated based on the following formula:

$$E=\left[10^{\left(-1/\text{slope}\right)-1}\right]\times 100\;\text{ranged from}\;90\%\text{to}\;110\%.$$

#### 2.5.3 Quantitative PCR analysis

qPCR was performed in duplicate for each cDNA sample in 0.2‐mL qPCR strip tubes with optical caps (Gunster Biotech) using CFX96TM real‐time PCR detection system (Bio‐Rad, USA). HeraPlus SYBER Green qPCR kit (Willowfort, UK) was used for the qPCR in a total volume of 20 μL. A no‐template PCR control for non‐specific amplification was included in each run. The cycling thermal profiles were an initial step at 95°C for 3 min followed by 40 cycles of denaturation at 95°C for 10 s and annealing/extension at 60°C for 1 min. Fluorescence data were collected during the extension step. A melting curve analysis (default settings, 65–95°C: increment 0.5°C for 0.05 s) was run at the end of each PCR to assess the specificity of amplified products. Each primer pair was proven to have a single peak on the dissociation curve, and no primer dimer was observed in the no‐template control. The cycle threshold (Ct) value was determined using the automatic settings on the CFX96TM real‐time PCR detection system.

### 2.6 Data analysis

Fold change in genes expression was calculated using the Pfaffl (2001) method. The normalization factor of gene expression was calculated based on the geometric mean of the two housekeeping genes (*β‐actin* and *GAPDH*). Data are presented as mean ± standard error of the mean (SEM). Graph‐Pad Prism (GraphPad 8.0.1 Software, San Diego, CA, USA) was used for statistical analysis and figure construction. Significant differences were analysed using two‐way analysis of variance (ANOVA); *p* < 0.05 was considered significant. Tukey's multiple range test was used for the pair‐wise comparison of means.

### 2.7 Histological and pathological examination

Fresh samples from the head kidneys, spleen and gills of both control and infected fish (*n* = 3/group/time point) were immediately fixed in 10% neutral‐buffered formalin solution for 48 h, then prepared using conventional histological processing and paraffin embedding techniques. Consequently, paraffin sections, 4 μm thick, were stained with haematoxylin and eosin (H&E) for histological examination using light microscopy, previously described by Drury and Wallington (1980).

## 3 RESULTS

### 3.1 Gross pathology and mortality rate

Upon arrival at the aquatic animal housing facility, preliminary disease screening of *C. gariepinus* from the first batch did not reveal any significant external parasitic infestations. However, *Chilodonella* was detected at a very low rate, approximately 0–1 per slide, which was considered a normal parasitic burden in pond‐reared catfish. With the stress resulting from fish handling and transportation, *Chilodonella* spp. multiplied in numbers and caused an acute infection, which was severe 3 days PT. Fish were off‐feed, irritable and displayed typical signs of external parasitic infection, such as flash swimming, skin depigmentation, widespread haemorrhages and ulcerations, fin erosion and gill erosions (Figure S2). Despite the salt treatment administered on day 5 PT, mortalities started in the infected group on day 7 PT, and the cumulative mortality rate reached 62.2% on day 13 PT (Figure S3). However, the infection rate/burden varied over the duration of the study. From day 13 onwards, the surviving infected fish were emaciated but displayed less‐severe clinical signs that faded away over time until the end of the study (28 days PT). The control uninfected fish exhibited no clinical signs of infection, showed normal behaviour and experienced no mortality throughout the study.

### 3.2 Parasite detection and quantification

Microscopical examination of the infected fish revealed a ciliate ectoparasite infection on the gills, skin and fins. The protozoan parasite was identified as *Chilodonella hexasticha* based on the morphological characteristics (body dimensions and kineties arrangement, Figure S4) according to Lom and Dyková (1992). Gills showed heavier parasitic loads compared to the skin and fins. On the day 7 PT, gills contained significantly higher numbers of *C. hexasticha*. On days 14‐ and 28 PT, the infected fish contained low parasitic numbers. The control fish examined at each time point remained negative for *C. hexasticha* infection throughout the study. A quantitative scoring of the severity of infection in gills at various sampling points is presented in Table S2.

### 3.3 Expression of immune‐relevant genes

**FIGURE 1:**
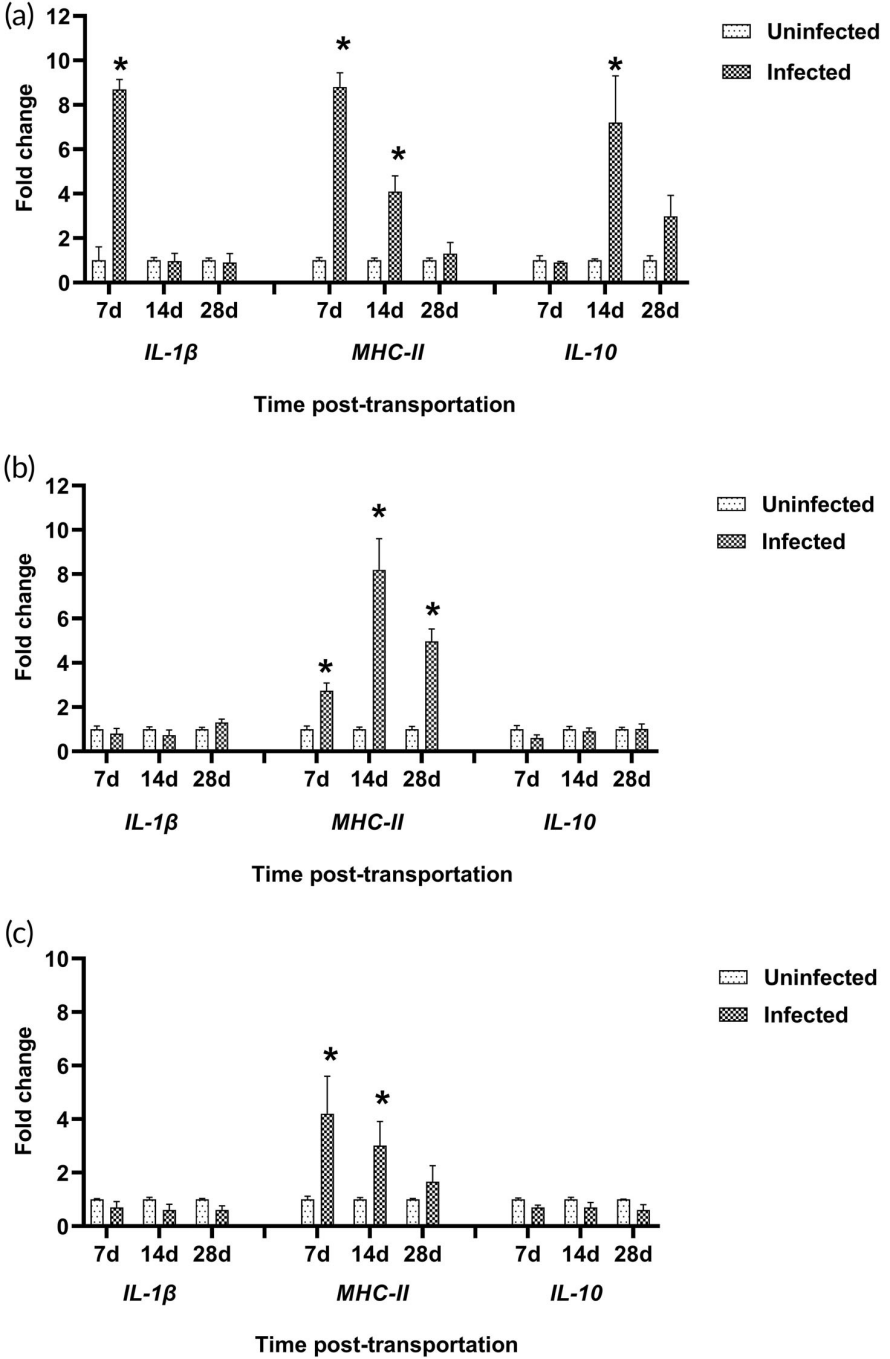
Gene expressions of *IL‐1β*, *MHC‐II* and *IL‐10* in *Clarias gariepinus* naturally infected with *Chilodonella hexasticha*. Expression of gills (a), head kidneys (b) and spleen (c) on days 7‐, 14‐ and 28 post‐transportation. Data represent mean expression levels ± standard error of the mean (SEM) (*n* = 3/group/time point). Bars labelled with asterisks were significantly different when compared to corresponding control [two‐way analysis of variance (ANOVA) test; *p* < 0.05] within a given time point.

**FIGURE 2:**
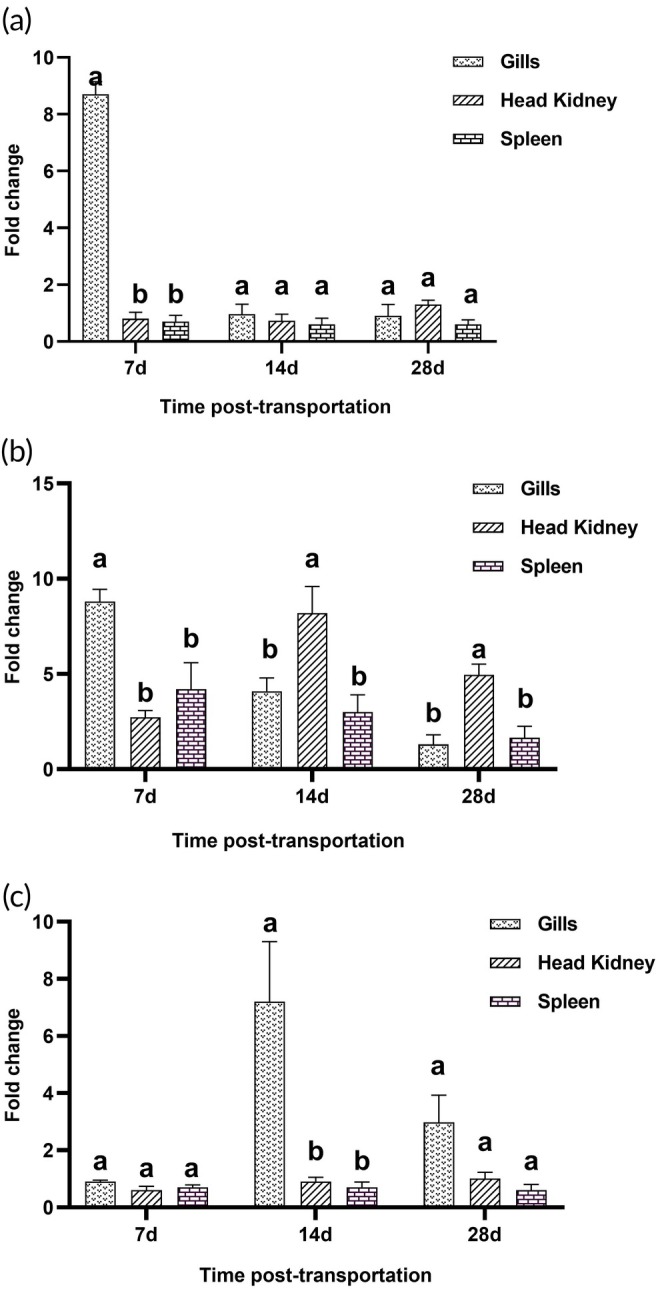
Comparison of *IL‐1β*, *MHC‐II* and *IL‐10* expression in the gills, head kidneys and spleen. Tissue expression profiles in terms of fold changes for *IL‐1β* (a), *MHC‐II* (b) and *IL‐10* (c) in *Clarias gariepinus* naturally infected with *Chilodonella hexasticha* on days 7‐, 14‐ or 28 post‐transportation, compared to uninfected fish. Data represent mean expression levels ± standard error of the mean (SEM) (*n* = 3/group/time point). Bars labelled with different lowercase letters were significantly different [two‐way analysis of variance (ANOVA) test; *p* < 0.05] within a given time point.

All gene expression results are shown in Figures 1 and 2. Significant upregulation (*p* < 0.05) of *IL‐1β* (8.3‐fold change) was observed in gill tissues of *Chilodonella*‐infected fish on day 7 PT when compared to the uninfected controls (Figure 1a). In contrast, *IL‐1β* expression levels showed no significant changes in head kidneys or spleen on days 14 and 28 PT (*p* > 0.05; Figure 1b, c).

The gene encoding *MHC‐II* was significantly upregulated (*p* < 0.05) in the gills and spleen on days 7 (9.1‐ and 4.2‐fold changes, respectively) and 14 PT (4.1‐ and 3‐fold changes, respectively) when compared to the uninfected control (Figure 1a, c). Similarly, *MHC‐II* was significantly upregulated (*p* < 0.05) in the head kidneys on days 7‐, 14‐ and 28 PT (2.8‐, 8.3‐ and 4.95‐fold change, respectively; Figure 1b).

The only significant change (*p* < 0.05) observed in the gene encoding *IL‐10* was upregulation (6.8‐fold change) in the gills on day 14 PT. The expression levels of this gene were higher in the gills of infected *C. gariepinus* compared to those in the central immune organs (Figure 1a–c).

The *IL‐1β* and *MHC‐II* genes reached their maximum expression levels in the gills on day 7 PT compared to the other tissues. *MHC‐II* gene expression was higher in the head kidneys compared to the gills and spleen on days 14 and 28 PT. On day 7 PT, *IL‐10* expression was similar in the gills, head kidneys and spleen. The maximum expression of *IL‐10* was observed in the gills on day 14 PT, and the gills also had the highest expression by day 28 PT (Figure 2a–c).

### 3.4 Histological and pathological findings

**FIGURE 3:**
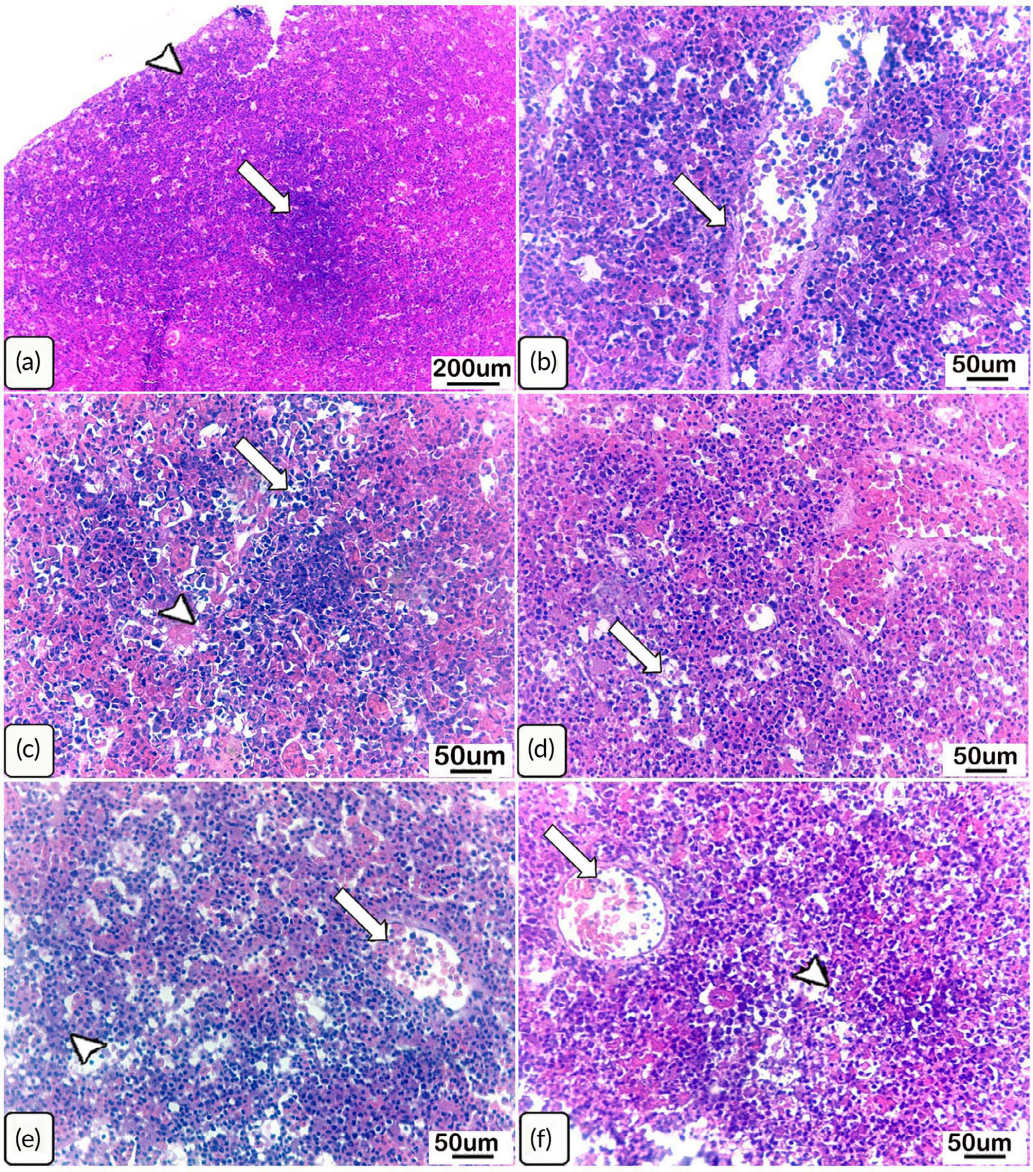
Light photomicrographs of haematoxylin and eosin (H&E)‐stained head kidney sections from control (uninfected) *Clarias gariepinus* and from those naturally infected with *Chilodonella hexasticha*. (a) Uninfected head kidney showing intact haematopoietic tissues (arrow) and a healthy capsule (arrowhead). (b) Infected head kidney at day 7 post‐transportation showing clearly detectable congestion of the blood vessels (arrow). (c) Infected head kidney on day 7 post‐transportation showing reduction in number of haematopoietic cells (arrow), in addition to mildly stagnant blood vessels (arrowhead). (d) Infected head kidney on day 14 post‐transportation showing moderately depleted haematopoietic tissues replaced by clear vacuoles (arrow). (e) Infected head kidney on day 14 post‐transportation showing depletion of the haematopoietic cells (arrowhead) and expanded blood vessels (arrow). (f) Infected head kidney on day 28 post‐transportation showing congestion of the blood vessels (arrow) and also a mild reduction in the haematopoietic cells (arrowhead). Scale bars = 50 and 200 μm, *n* = 3/group/time point.

Histological sections of uninfected *C. gariepinus* head kidneys exhibited intact architecture of the haematopoietic tissues and healthy vasculature (Table 1; Figure 3a). On the contrary, examination of the head kidney tissues in the *Chilodonella*‐infected *C. gariepinus* on day 7 PT revealed remarkable congestion of the blood vessels and depleted haematopoietic tissues, which were identified by clear vacuolization (Table 1; Figure 3b,c). Only moderate changes were recorded on day 14 PT, which comprised moderate depletion of the haematopoietic cells and congestion of the blood vessels (Table 1; Figure 3d,e). Furthermore, the head kidney sections from the fish infected on day 28 PT showed histological changes in the haematopoietic tissues and blood vessels but to a lesser degree (Table 1; Figure 3f).

**TABLE 1:** Scoring of pathological changes in the gills of *Clarias gariepinus* infected with *Chilodonella hexasticha* and alterations in head kidneys, spleen and gills due to immune response to infection.

Changes	Uninfected	Days of infection post‐transportation		
	Control	Day 7	Day 14	Day 28
I. Head kidneys				
Melanomacrophage activation	+	++	+	+
Depletion of haematopoietic tissues	−	+++	++	+
Congestion and thickening of the blood vessels	−	+++	++	+
II. Spleen				
Activation of melanomacrophage centres	+	+++	+++	++
Depletion of haematopoietic tissues in red pulps	−	++	++	+
Thickening of splenic capsules	−	++	+	−
Thickening of the blood vessel walls	−	+++	+	−
Congestion and dilatation of the blood vessels	−	+++	+	+
III. Gills				
Lamellar necrosis and vacuolation	−	+++	+++	+
Sloughing and desquamation of epithelium	−	+++	++	+
Shortening of the secondary lamellae	−	+++	++	+
Thickening and hyperplasia of the lamellae	−	+++	++	+
Fusion of secondary lamellae	−	+++	++	++
Mononuclear cell infiltration	+	+++	+++	+
Congestion and dilatation of the blood vessels	−	+++	++	+

*Note*: (−), not detectable; (+), mild; (++), moderate; (+++), severe. *n* = 3/group/time point.

**FIGURE 4:**
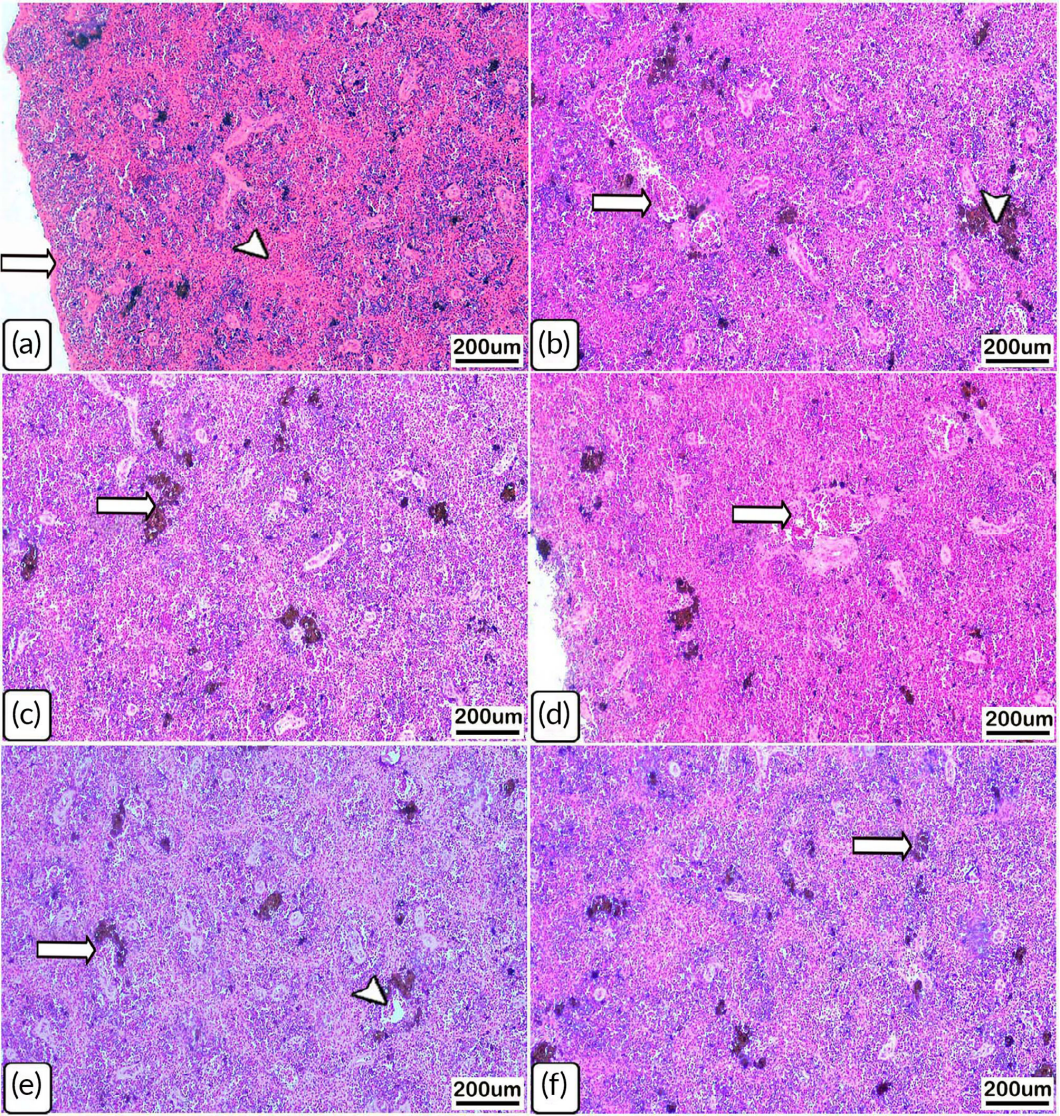
Light photomicrographs of haematoxylin and eosin (H&E)‐stained spleen sections from control (uninfected) *Clarias gariepinus* and from those naturally infected with *Chilodonella hexasticha*. (a) Uninfected spleen showing intact splenic pulps (arrowhead) enclosed by healthy capsule (arrow). (b) Infected spleen on day 7 post‐transportation showing congestion of splenic vasculature (arrow) with melanomacrophage infiltration (arrowhead). (c) Infected spleen on day 7 post‐transportation showing scattered infiltration of melanomacrophage cells (arrow). (d) Infected spleen on day 14 post‐transportation showing minimally congested and thickened blood vessels (arrow). (e) Infected spleen on day 14 post‐transportation showing melanomacrophage cells (arrow) and slightly congested vessels (arrow head). (f) Infected spleen on day 28 post‐transportation showing infiltration of melanomacrophages (arrow). Scale bars = 200 μm, *n* = 3/group/time point.

The histological sections from the uninfected catfish spleens revealed intact splenic capsules and pulps (Table 1; Figure 4a). In contrast, the spleen sections from infected *C. gariepinus* on day 7 PT contained significant infiltration of melanomacrophages and exhibited depletion of haematopoietic cells in the red pulps and congestion of the splenic sinusoids (Table 1; Figure 4b,c). Slightly congested splenic vessels and infiltration by melanomacrophages were also observed on day 14 PT (Table 1; Figure 4d,e). Similar to the head kidney tissues on day 28 PT, the spleens had ill‐defined histological alterations, which predominantly consisted of melanomacrophage infiltration (Table 1; Figure 4f).

**FIGURE 5:**
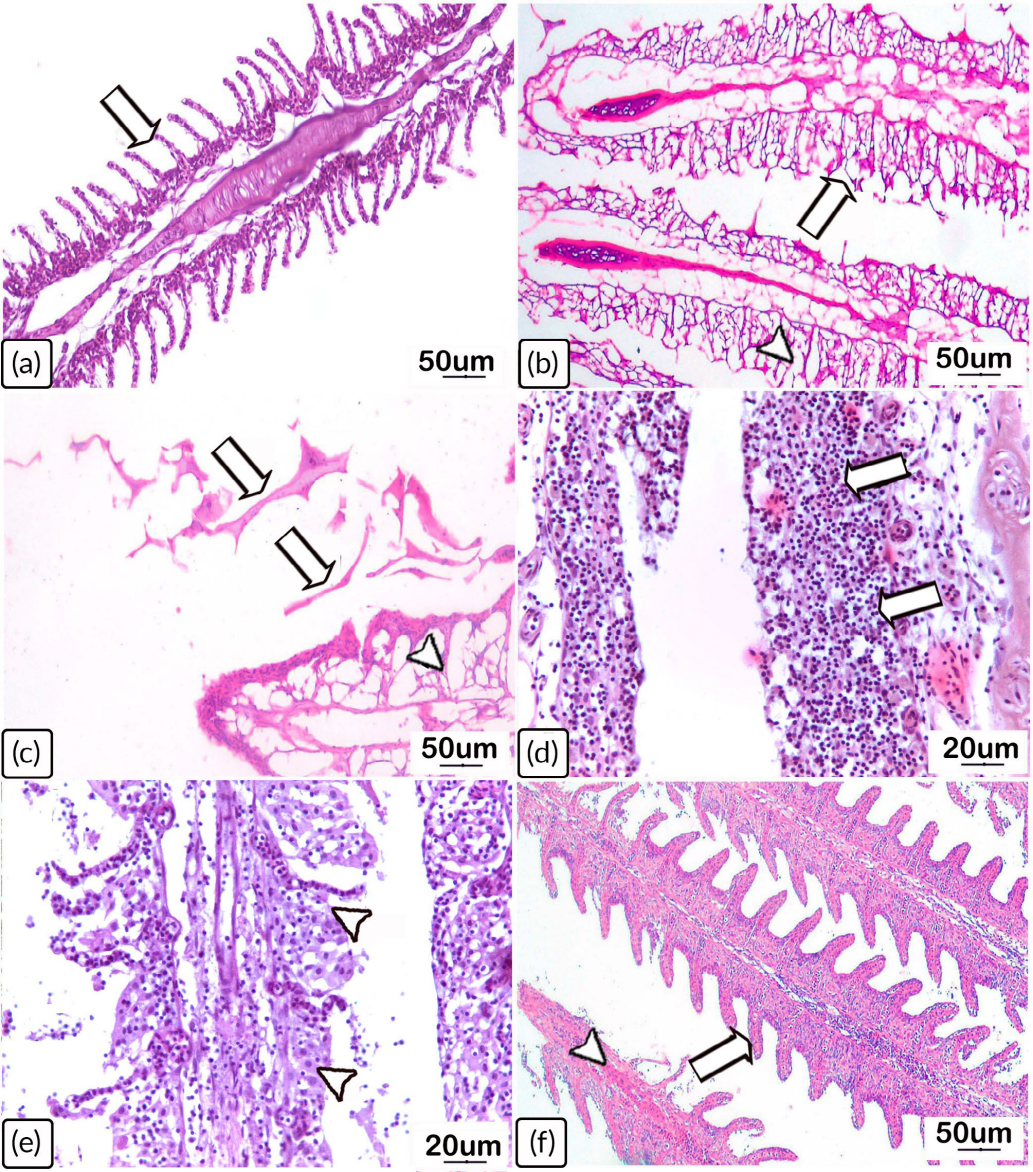
Light photomicrographs of haematoxylin and eosin (H&E)‐stained gill sections from control (uninfected) *Clarias gariepinus* and from those naturally infected with *Chilodonella hexasticha*. (a) Uninfected gills showing normal architecture of gill tissues (arrow). (b and c) Gills on day 7 post‐transportation (PT) showing necrosis and sloughing of lamellar tissues (arrows) and vacuolization of the gill lamellae compensating for necrosed tissue (arrowheads). (d) Infected gills on day 7 PT showing desquamated gill tissues differentiated by a very large infiltration of leukocytes (arrow). (e) Gills on day 14 PT showing focal aggregation of inflammatory cells replacing necrotic and desquamated tissue (arrowhead). (f) Gills on day 28 PT showing a mild degree of lamellar hyperplasia (arrow) and a slightly congested central venous sinus (arrowhead). Scale bars = 50 and 20 μm, *n* = 3/group/time point.

Observation of the gills from the uninfected *C. gariepinus* revealed normal arrangements and structures of the primary and secondary lamellae (Table 1; Figure 5a). Intense lamellar vacuolization and necrosis were detected in the gill parenchyma from fish infected on day 7 PT (Table 1; Figure 5b,c). In addition, there were dissemination and infiltration of inflammatory cells, representing severe necrosis and desquamation, where leukocytes were predominantly detected (Table 1; Figure 5d). By day 14 PT, the *Chilodonella* infection load in the gills resulted in discrete necrosis with sloughing of the gill lamellae, with heavy accumulation of mononuclear infiltrates (Table 1; Figure 5e). On day 28 PT, a mild level of histological deterioration was recorded. This was visualized as blood capillary congestion, mononuclear infiltration and scattered proliferation of the epithelial lining of the gill lamellae accompanied by epithelial thickening and lamellar hyperplasia (Table 1; Figure 5f).

## 4 DISCUSSION

The aim of this study was to explore the immune responses of *C. gariepinus* to a natural external infection with *C. hexasticha*, a ciliate protozoan parasite, following transportation stress. The present study investigated the responses of *C. gariepinus* to chilodonellosis by examining the expression of certain innate and adaptive immune‐related genes (*IL‐1β*, *MHC‐II* and *IL‐10*) in the gills, head kidneys and spleen to determine whether the immune response to the infection was localized to the gills or systemically extended beyond the external sites of infection to the central immune organs.

Chilodonellosis is a serious disease facing the fish farming industry and causes great economic losses (Li et al., 2018). Infections with *Chilodonella* spp. can be provoked by stressful conditions (Paperna & Van As, 1983) or rapid changes in environmental parameters (Bowater & O'Donoghue, 2015). Results of this study demonstrate that stress associated with routine fish transportation can serve as a potent trigger for opportunistic parasitic infections, such as *C. hexasticha*, even in fish that initially appear healthy. This was supported by Sutthi and Doan (2020) who suggested that the stress fish are exposed to during transport can suppress immune system functions compromising their ability to combat infection and increasing their susceptibility to diseases and mortalities. In the present study, although the fish were apparently healthy at the farm, they may have been suffering from subclinical or trivial *Chilodonella* infestation with masked signs as previously suggested by Noga (2010) who stated that fish infected with small numbers of *Chilodonella* rarely exhibit disease symptoms unless stressed. Transporting those fish may have predisposed them to immunosuppression and flourishing of the parasite that led to the onset of clinical *Chilodonella* infection with manifested signs shortly after transportation (3 days).

In this study, natural infection with *C*. *hexasticha* was not initially anticipated, as the fish were obtained for a different research project. Therefore, tracking the onset of the infection from the beginning (day 1) was not feasible. However, as fish mortalities started on day 7 PT, samples were collected on days 7‐, 14‐ and 28 PT of the *C. gariepinus* to the aquatic animal housing facility. *Chilodonella* epizootics at this farm site have been repeatedly reported on an annual basis from late summer through early winter depending on environmental factors such as the water temperature (personal communication with the fish farmer).

Results of this study revealed that *C. gariepinus* responded to *Chilodonella* infection at the external site of infection (gills) and also at the central immune organs (head kidneys and spleen) with differential expression of both innate and adaptive immune genes. Moreover, the magnitude of fish's immune reactions in response to the infection was correlated to the severity of *Chilodonella* infection, which was highest on day 7 PT. *Chilodonella* can rapidly multiply and double their numbers in only a few hours (Pádua et al., 2013), and the antigenic presentation from the growth of the parasites can also stimulate responses in the infected tissues (Syahputra et al., 2019).

Several studies reported upregulation of cytokine expression in fish in response to various protozoal parasitic groups (Bridle et al., 2006; Morrison et al., 2007), monogenea (Faliex et al., 2008), digenea (Sandor et al., 2003) and copepoda (Covello et al., 2009; Fast et al., 2006). IL‐1β, one of the earliest expressed proinflammatory cytokines (Reyes‐Cerpa et al., 2013), helps the migration of neutrophils and macrophages to the infection site (Secombes et al., 2001) and enables the host to react quickly to infections by triggering a chain of events that results in inflammation (Reyes‐Cerpa et al., 2013). In this study, the significantly high level of *IL‐1β* expression in the gills of fish infected with *Chilodonella*, noticed on day 7 PT, correlated with the highest parasite load present in the gill tissues at this time point, indicating maximum colonization. Unsurprisingly, the gill tissues showed significantly higher levels of *IL‐1β* expression than those in the head kidneys and spleen, especially at the earliest stage of infection (on day 7 PT), which could be due to the localization of the parasite exclusively in gills and at the external surfaces. Moreover, the gill tissue is a primary site of constitutive *IL‐1β* expression, as the specialized mucosa‐associated immune cell aggregations (gill‐associated lymphoid tissues) are located within the gills (Buchmann, 2020) and are known to be cytokine‐synthesizing cells. Upregulated expression of *IL‐1β* in the gills may provide defence at the port of entry and permit a rapid localized immune response to *Chilodonella*. Syahputra et al. (2019) reported that the gills responded significantly stronger compared to skin following an experimental infection of *Oncorhynchus mykiss* with the ciliated protozoan *I. multifiliis*.

In contrast to the *IL‐1β* results, the response in the head kidneys and spleen, which are not directly exposed to *Chilodonella* infection, was mostly directed towards *MHC‐II* gene regulations. *MHC‐II* mainly presents exogenously derived peptides to CD4^+^ T cells (Grimholt et al., 2003). In the present study, the upregulation of *MHC‐II* levels in the spleen and gills on days 7‐ and 14 PT, as well as in the head kidneys at all time points studied, may result from proinflammatory cytokine stimulation. *IL‐1β* has the capacity to induce macrophages to express *IL‐1* and *MHC‐II* (Hong et al., 2001). This indicates that local protecting responses in the mucosal surface (gills) could be transferring signals to the central immune organs, triggering a systemic response a few days following the initial infection through migrating leucocytes. This is supported by the findings of this study, showing that expression of *MHC‐II* in the gills was significantly higher than that in the central immune organs earlier in the infection (on day 7 PT), whereas the expression became significantly higher in the head kidneys later on days 14 and 28 PT. These results imply that the head kidneys are a more active lymphoid internal organ for regulating cellular immune reactions during infection. Therefore, localized reactions in the mucosal tissues in combination with the adaptive responses in central immune organs become important actions that direct systemic immunity (Buchmann, 2020).

Results from this study showed that the modulatory cytokine *IL‐10* was significantly upregulated in the gills on day 14 PT, which corresponded to the fading away of the infection and, consequently, downregulation of *IL‐1β*. Fish *IL‐10* has been confirmed to be anti‐inflammatory, suppressing phagocytosis and expression of proinflammatory cytokines (Grayfer et al., 2011). This could help control proinflammatory responses during infection (Munoz et al., 1991), restricting the potentially injurious actions elicited by excessive inflammation (Opal & DePalo, 2000). This is also supported by the findings in the present study, showing that there was gradual alleviation of clinical signs and mortality over the second week of the infection, alongside the return of *IL‐1β* gene expression levels to near the control values in the gills. Therefore, after the generation of proinflammatory immune response, *IL‐10* acts to inhibit any excessive or prolonged inflammation that could be deleterious to the host itself, reducing potential tissue damage as indicated by the histopathological findings on day 28 PT.

The central immune organs exhibited histological changes resulting from systemic immune responses to the infection, along with changes in the cellular activities that were potentially manifested by altered gene expression. Internally, the histological alterations observed in the head kidneys and spleens of the *Chilodonella*‐infected *C. gariepinus* may result from immune responses triggered by the processing of parasite antigens and debris. In teleosts, antigen‐presenting cells in the gill epithelium (Kato et al., 2018), including dendritic cells or macrophages, can internalize parasitic antigens at the surface, then migrate to systemic immune organs, to stimulate adaptive immunity against parasites (Buchmann, 2020), which is further supported by the gene expression findings in this study.

The primary histopathological alterations induced by *Chilodonella* were mainly noticeable in the gills due to its abrasive action on the host epithelium, particularly on the gill filaments, which are the most sensitive organ in relation to parasitic attacks. Infection by *Chilodonella* causes severe histological lesions in comparison to infection by other ciliated trichodinids. The rapid replication and constant rotation of the parasite in the gills could explain the damage to gill structure and the severe inflammation noticed in infected fish on day 7 PT. Besides this, the gills of the *Chilodonella*‐infected fish demonstrated proliferation of the filament epithelium, necrosis, desquamation and mononuclear infiltrations, therefore, indicating cytokine‐secreting cells (Pádua et al., 2013).

## 5 CONCLUSIONS

The results of the present study demonstrate that *Chilodonella* pathogenesis in *C. gariepinus* is modelled by a profound mucosal and systemic immune response. *Chilodonella* pathogenesis in the gills begins with a phagocyte‐mediated proinflammatory response derived by increased *IL‐1β* expression and followed by a prevailing anti‐inflammatory response with an increase in the expression of *IL‐10*. In contrast, *Chilodonella* pathogenesis in the head kidneys and spleen was demonstrated by increased expression of the *MHC‐II* cell receptor initiating an adaptive immune response. These responses help the fish counteract the parasitic infections that were possibly exacerbated by transportation stress. This underlines the biological significance of transportation stress as an immunomodulatory factor and suggests that fish health management strategies should be deployed with fish transport. Implementing best practices, monitoring the immune status and screening for low‐level parasitic burdens prior to and after transport can significantly reduce disease outbreaks and associated economic losses in aquaculture. Furthermore, the results showed structural changes in both mucosal and central immune organs, which may be due to the physiological impact of stress, infection and response to the infection.

## AUTHOR CONTRIBUTIONS

All authors contributed to the work stated, whether in the development, planning and implementation; data acquisition, analysis and comprehension; or in all of these areas; participated in writing, editing or critically reviewing the article; provided the final authorization of the version to be published; agreed on the journal to which the article was submitted; and agreed to be accountable for all aspects of the work.

## FUNDING INFORMATION

Private donation funding support and University of Nottingham.

## CONFLICT OF INTEREST STATEMENT

The authors declare no conflicts of interest.

## Supporting information


**Figure S1.** Schematic protocol summary of the experimental protocol. *Clarias gariepinus* were allocated into two groups: the first group served as *Chilodonella hexasticha*‐infected group, and the second group served as the uninfected controls. Tissue samples were collected on days 7‐, 14‐ and 28 post‐transportation from both the infected and control groups from the skin and gills for parasitological examination, and from the gills, head kidneys and spleen for gene expression and histopathology analysis.

**Figure S2.** Gross anatomical photographs show that *Clarias gariepinus* naturally infected with ciliated *Chilodonella hexasticha*, showing haemorrhages in the head (a), skin ulceration and haemorrhages (b), fin and tail haemorrhages and sloughing (c) and erosion of gills (d), as demonstrated by the arrows.

**Figure S3.** Cumulative mortality percentage of *Clarias gariepinus* naturally infected with *Chilodonella hexasticha*.

**Figure S4.** *Chilodonella hexasticha* smears and staining in *Clarias gariepinus* gills. (a) Light photomicrographs of fresh unstained smears of *C. hexasticha*. (b) Giemsa‐stained *C. hexasticha* specimen. Ci, Cilia; cyt, cytopharynx; Lk, left kineties; Ma, macronucleus; Rk, right kineties. Scale bars represent 100 and 20 μm, respectively.

**Table S1.** Primers designed for this study and used for real‐time polymerase chain reaction (PCR) for the detection of *Clarias gariepinus* target genes.
**Table S2.** Severity of infection of *Chilodonella hexasticha* in gills of *Clarias gariepinus*.

## Data Availability

The data that support the findings of this study are available from the corresponding author (WE) upon reasonable request.
