# Supplementary figures and images for: Insights into mucosal and systemic immune responses of African catfish, Clarias gariepinus, to chilodonellosis: A natural infection study
Source: J Fish Biol. 2025 Jun 30;107(4):1323–34. doi: 10.1111/jfb.70131 (PMC12536051; doi:10.1111/jfb.70131)

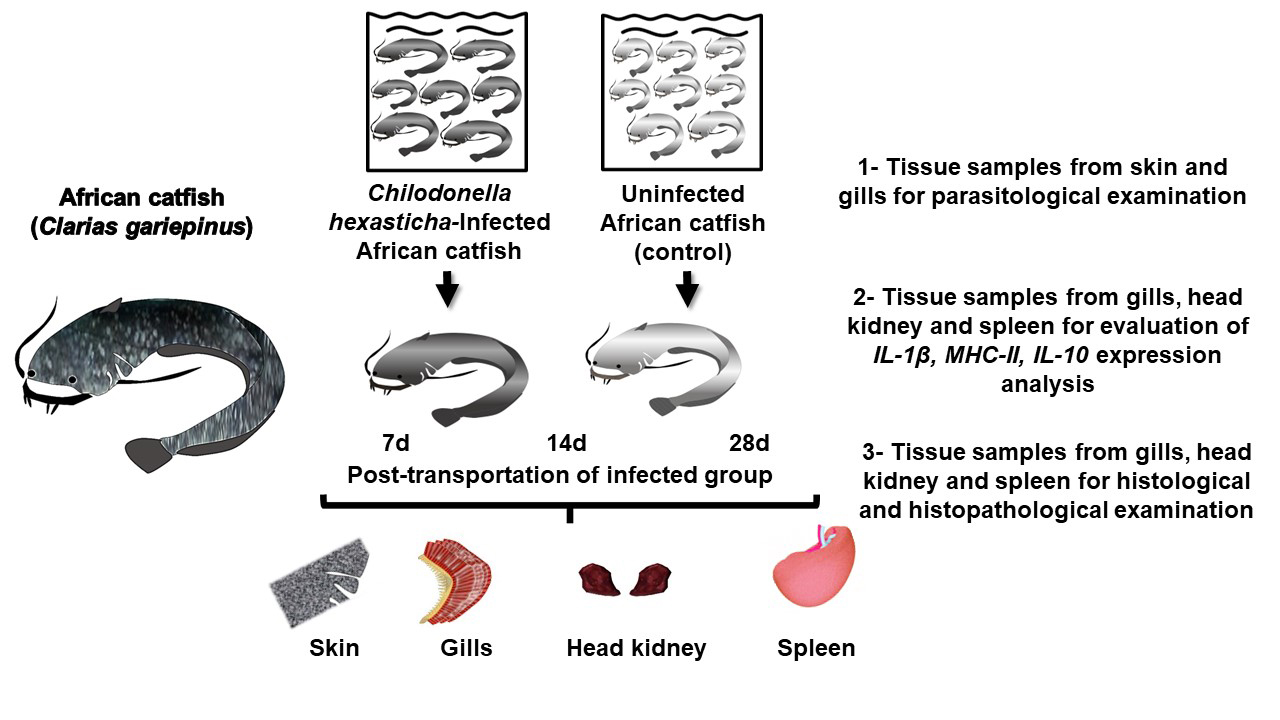

Supplement: Supplementary file 1 — Figure S1. Schematic protocol summary of the experimental protocol. Clarias gariepinus were allocated into two groups: the first group served as Chilodonella hexasticha‐infected group, and the second group served as the uninfected controls. Tissue samples were collected on days 7‐, 14‐ and 28 post‐transportation from both the infected and control groups from the skin and gills for parasitological examination, and from the gills, head kidneys and spleen for gene expression and histopathology analysis. [file JFB-107-1323-s005.jpg]

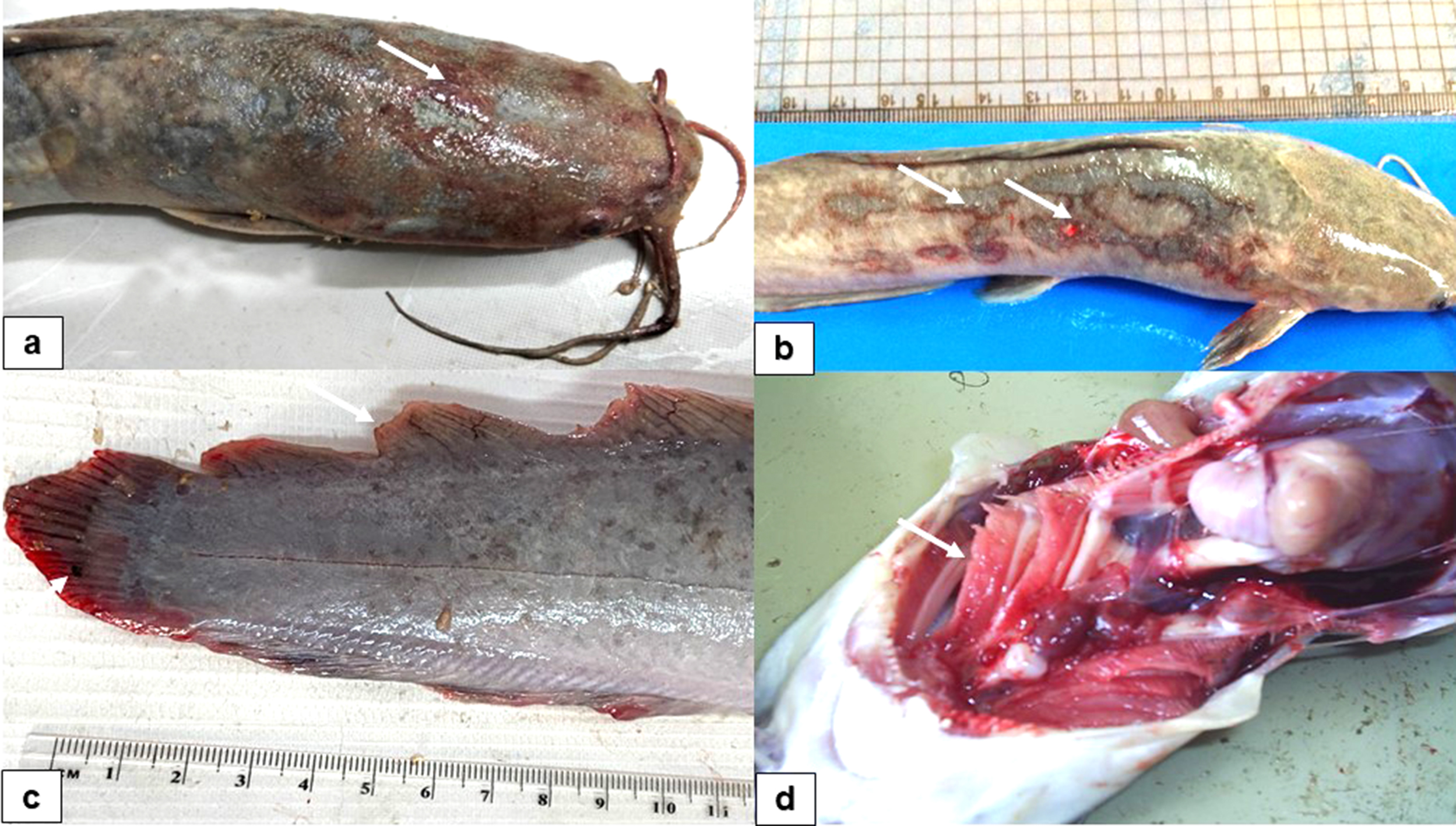

Supplement: Supplementary file 2 — Figure S2. Gross anatomical photographs show that Clarias gariepinus naturally infected with ciliated Chilodonella hexasticha, showing haemorrhages in the head (a), skin ulceration and haemorrhages (b), fin and tail haemorrhages and sloughing (c) and erosion of gills (d), as demonstrated by the arrows. [file JFB-107-1323-s003.jpg]

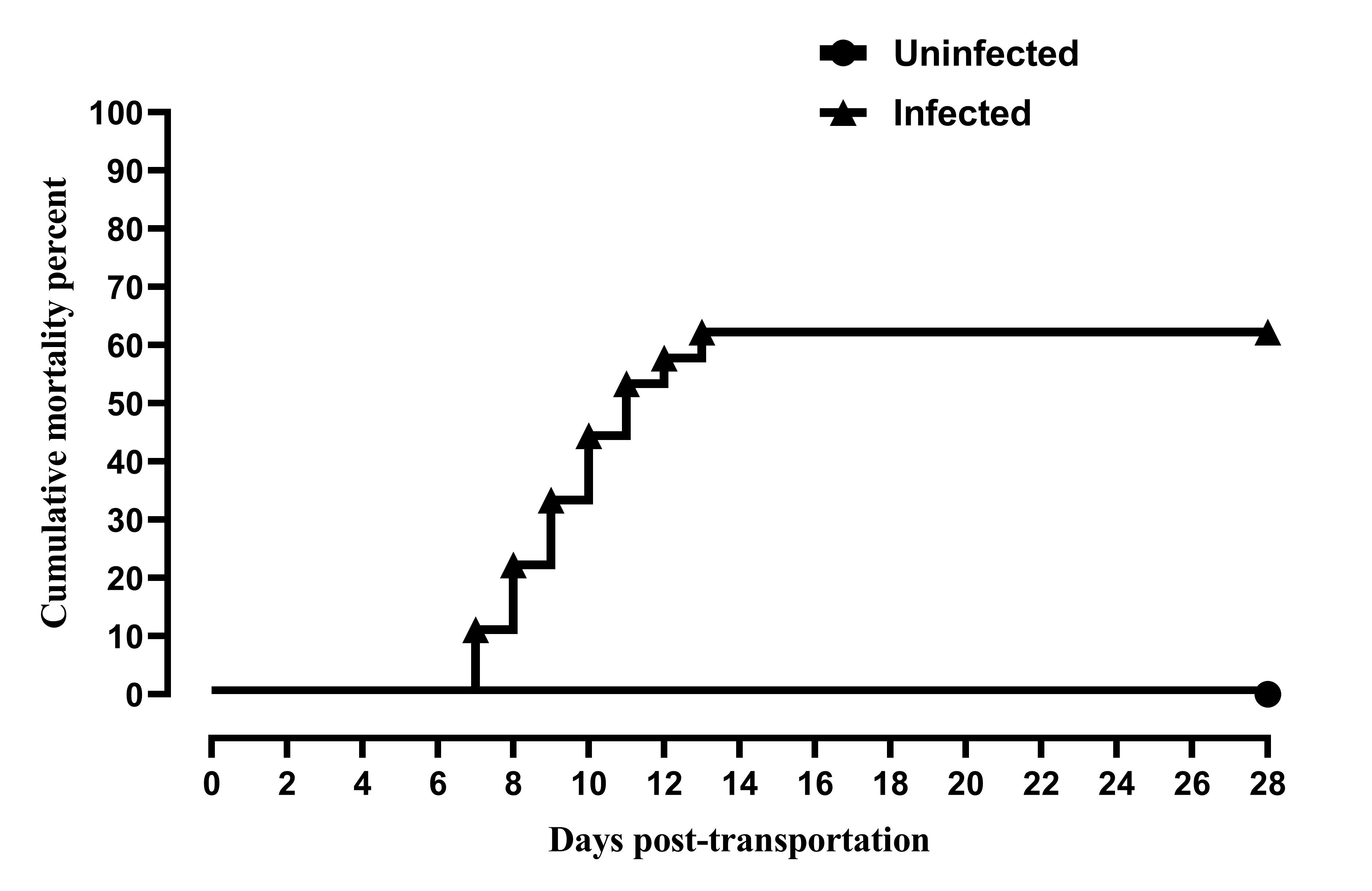

Supplement: Supplementary file 3 — Figure S3. Cumulative mortality percentage of Clarias gariepinus naturally infected with Chilodonella hexasticha. [file JFB-107-1323-s004.jpg]

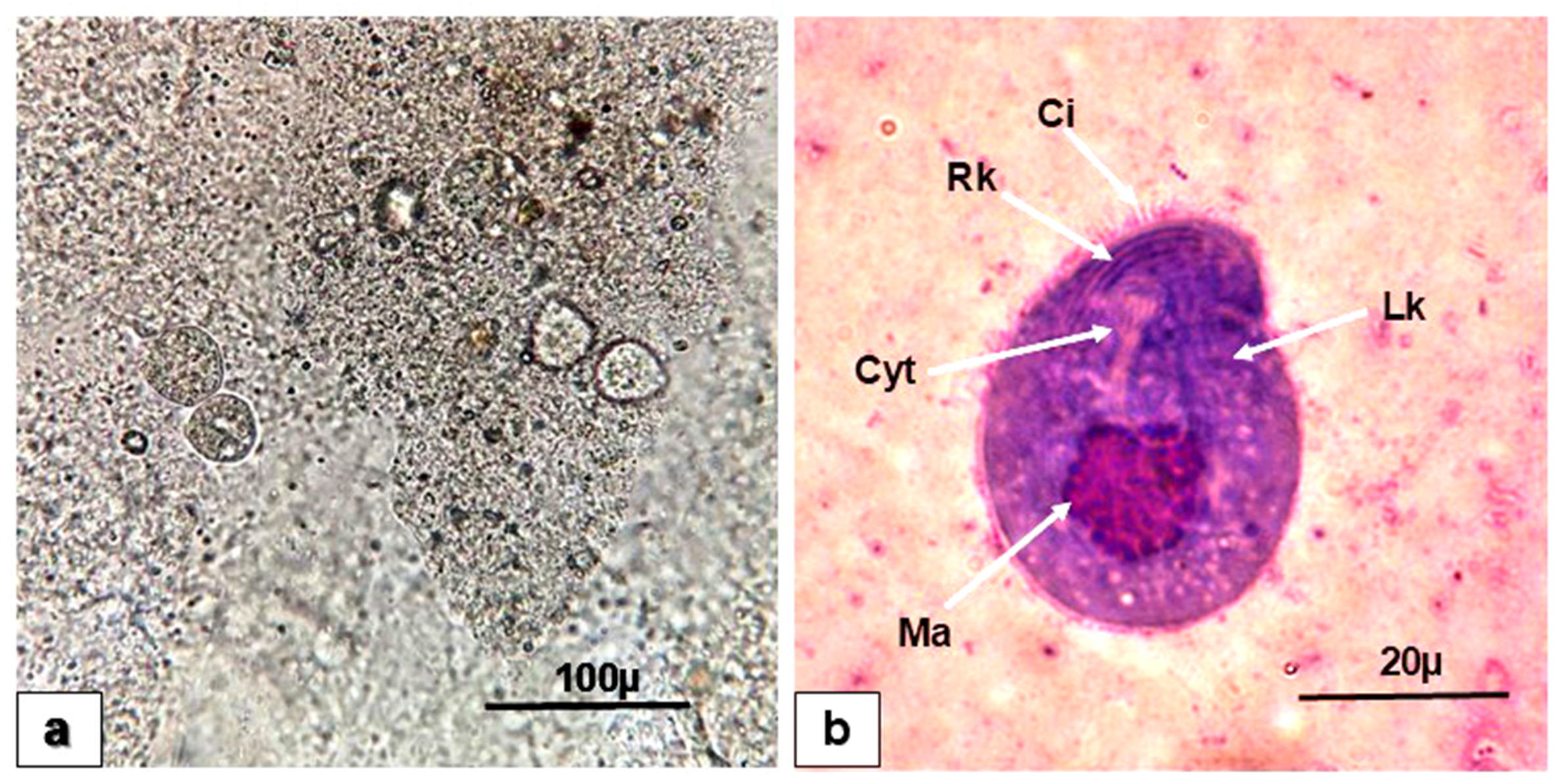

Supplement: Supplementary file 4 — Figure S4. Chilodonella hexasticha smears and staining in Clarias gariepinus gills. (a) Light photomicrographs of fresh unstained smears of C. hexasticha. (b) Giemsa‐stained C. hexasticha specimen. Ci, Cilia; cyt, cytopharynx; Lk, left kineties; Ma, macronucleus; Rk, right kineties. Scale bars represent 100 and 20 μm, respectively. [file JFB-107-1323-s002.jpg]
