# Supplementary material for: Insights into mucosal and systemic immune responses of African catfish, Clarias gariepinus, to chilodonellosis: A natural infection study
Source: J Fish Biol. 2025 Jun 30;107(4):1323–34. doi: 10.1111/jfb.70131 (PMC12536051; doi:10.1111/jfb.70131)
Supplement: Supplementary file 5 — Table S1. Primers designed for this study and used for real‐time polymerase chain reaction (PCR) for the detection of Clarias gariepinus target genes. Table S2. Severity of infection of Chilodonella hexasticha in gills of Clarias gariepinus. [file JFB-107-1323-s001.docx]

**Insights into mucosal and systemic immune responses of African catfish, *Clarias gariepinus*, to Chilodonellosis: A Natural Infection Study**

**Running title:** *Chilodonella*-infected African catfish

**Walaa F.A. Emeish^1,^*, Salwa Mansour^2^, Marwa M. Fawaz^3^, Ali H. Alghamdi^4^, Abdullah A. A. Alghamdi^5^, Zeinab Al-Amgad^6^, Haitham H. Mohammed^7,9^, Catrin S. Rutland^8,^*, Ahmad A. Elkamel^9^, Karima A. Bakry^1^**

^1^ Department of Fish Diseases, Faculty of Veterinary Medicine, South Valley University, Qena 83523, Egypt; <https://orcid.org/0000-0001-7523-3720>, <https://orcid.org/0000-0002-6609-2476>, ewalaa@vet.svu.edu.eg, karima-alaa@vet.svu.edu.eg

^2^ Zoology Department, Faculty of Science, South Valley University, Qena 83523, Egypt; <http://orcid.org/0000-0002-6682-178X>, salwa111986@sci.svu.edu.eg

^3^ Department of Parasitology, Faculty of Veterinary Medicine, South Valley University, Qena 83523, Egypt; https://[orcid.org/0000-0003-0560-9504](https://l.facebook.com/l.php?u=http%3A%2F%2Forcid.org%2F0000-0003-0560-9504%3Ffbclid%3DIwZXh0bgNhZW0CMTAAAR23e7Q3mtbIKIgd7u_T9RXmzU_QUIJLMdXzjXoLI3iCmR5-lDKpHD-WCV0_aem_AV7X2rGhJmhclSOCWKmFWPhtVE4BC0sjdFZY5jE8cKQci2-WquZV2z9_G4aczKd_zPZS68OSTkcnterLBzuysOD9&h=AT2fBCaGIfYxwntN2YluUfD_NNdALvoIrEbD0uvOK1f9su0IV1jHEX3YUmxYvgdJDp-8rFrep_UN0ZiXTYzWeh11O_GUu9hObd0e1iDCH5ONxj3ovWNcpwLAQEVSFNoFuYJU2g), m_abdallah@vet.svu.edu.eg

^4^ Department of Biology, Faculty of Science, Al-Baha University, Alaqiq, Saudi Arabia; <http://orcid.org/0000-0003-0426-9237>, [aayfan@bu.edu.sa](mailto:aayfan@bu.edu.sa)

^5^ Department of Biology, Faculty of Science, Al-Baha University, Al-Baha, Saudi Arabia; <https://orcid.org/0000-0003-3136-9081>, [aaa.alghamdi@bu.edu.sa](mailto:aaa.alghamdi@bu.edu.sa)

^6^ General Authority for Veterinary Services, Qena Veterinary Directorate, Qena 83511, Egypt; <https://orcid.org/0000-0002-4039-4759>, zizi_1283@yahoo.com

^7^ Department of Rangeland, Wildlife, and Fisheries Management, Texas A&M University, College Station, Texas 77843, USA; <https://orcid.org/0000-0002-0717-6055>, haitham.mohammed@ag.tamu.edu

^8^ School of Veterinary Medicine and Science, University of Nottingham, Nottingham, UK; catrin.rutland@nottingham.ac.uk

^9^ Department of Aquatic Animal Medicine and Management, Faculty of Veterinary Medicine, Assiut University, Assiut 71526, Egypt; <https://orcid.org/0000-0002-3319-7423>, [aelkamel@aun.edu.eg](mailto:aelkamel@aun.edu.eg)

***Correspondence:**

Catrin S. Rutland, catrin.rutland@nottingham.ac.uk

Walaa F.A. Emeish, ewalaa@vet.svu.edu.eg

**Supplementary File**

**Table 1s.** Primers designed for this study and used for real-time PCR for the detection of *Clarias gariepinus* target genes.

| Gene | Primer sequence (5`-3`) | GenBank Accession numbers | Amplicon size (bp) | Location of amplicon |
| --- | --- | --- | --- | --- |
| *GAPDH* | SEQ_F: GTGGAGCTAAGCGCGTGGTA  SEQ_R: GGGTGGCAGTGTAGGCATGG | AF323693 | 216 | 193-408 |
| *β-actin* | SEQ_F: GAGCTGCGTGTTGCCCCTGAG  SEQ_R: ACCGGAGTCCATCACAATACCAGT | XM_053492207 | 192 | 421-612 |
| *IL-1β* | SEQ_F: CGCTGGTGCAGCGTAACAAC  SEQ_R: TCACAATGCCCAGGCACACC | MH341527 | 147 | 131-277 |
| *MHC-II* | SEQ_F: AGCAGTCAGACGGCACTCAC  SEQ_R: CAGTGGACGTCACACCTCCC | MG545604 | 123 | 180-302 |
| *IL-10* | SEQ_F: TTGGACACGGTGTTGCCCAG  SEQ_R: TGTCCAGCTCTCCCATGGCT | MH341526 | 211 | 187-397 |

**Table 2s.** Severity of infection of *Chilodonella hexasticha* in gills of *Clarias gariepinus*.

| Time points post-transportation | Number of *C.* *hexasticha* | Degree of severity |
| --- | --- | --- |
| 7 days | 5-10 / microscopic field | Severe |
| 14 days | 3-4 / microscopic field | Moderate |
| 28 days | 1-2 / microscopic smear | Mild |
| Uninfected control group | 0 | None detected |

One smear per fish on days 7, 14 and 28, with 5 fields per smear.

**Figure legends**

**Figure 1s.** Schematic protocol summary of the experimental protocol. *Clarias gariepinus* were allocated into two groups, the first group served as *Chilodonella* *hexasticha*-infected group, and the second group served as the uninfected controls. Tissue samples were collected at 7-, 14- and 28-days post-transportation from the infected and control groups from skin and gills for parasitological examination, and from the gills, head kidneys and spleen for gene expression and histopathology analysis.

**Figure 2s.** Gross anatomical photographs show *Clarias gariepinus* naturally infected with ciliated *Chilodonella* *hexasticha*. Showing hemorrhages in the head (a), skin ulceration and hemorrhages (b), fin and tail hemorrhages and sloughing (c), and erosion of gills (d), as demonstrated by the arrows.

**Figure 3s.** Cumulative mortality percent of *Clarias gariepinus* naturally infected with *Chilodonella hexasticha.*

**Figure 4s.** *Chilodonella hexasticha* smears and staining in *Clarias gariepinus* gills. (a) Light photomicrographs of fresh unstained smears of *Chilodonella hexasticha.* (b) Giemsa-stained *Chilodonella hexasticha* specimen. (Ci) Cilia, (cyt) cytopharynx, (Lk) Left kineties, (Ma) macronucleus, (Rk) Right kineties. Scale bars represent 100 & 20 µm, respectively.
